# Low Computational-Cost Footprint Deformities Diagnosis Sensor through Angles, Dimensions Analysis and Image Processing Techniques

**DOI:** 10.3390/s17112700

**Published:** 2017-11-22

**Authors:** J. Rodolfo Maestre-Rendon, Tomas A. Rivera-Roman, Juan M. Sierra-Hernandez, Ivan Cruz-Aceves, Luis M. Contreras-Medina, Carlos Duarte-Galvan, Arturo A. Fernandez-Jaramillo

**Affiliations:** 1 Unidad Académica de Ingeniería Biomédica, Universidad Politécnica de Sinaloa, Carretera Municipal Libre Mazatlán Higueras km 3, Col. Genaro Estrada, Mazatlán Sin. 82199, Mexico; jmaestre@upsin.edu.mx (J.R.M.-R.); 2015030124@upsin.edu.mx (T.A.R.-R.); 2 Center for Biomedical Technology, Polythecnic University of Madrid, Campus Montegancedo, Pozuelo de Alarcón, Madrid 28223, Spain; 3 Departamento de Ingeniería Electrónica, División de Ingenierías, Universidad de Guanajuato, Carretera Salamanca-Valle de Santiago km 3.5 + 1.8, Comunidad de Palo Blanco, Salamanca Gto. C.P. 36885, Mexico; jm.sierrahernandez@ugto.mx; 4 CONACYT, Centro de Investigación en Matemáticas (CIMAT), A.C., Jalisco S/N, Col. Valenciana, Guanajuato Gto. 36000, Mexico; ivan.cruz@cimat.mx; 5 CA Ingeniería de Biosistemas, División de Investigación y Posgrado, Facultad de Ingeniería, Universidad Autónoma de Querétaro, Cerro de las campanas S/N, Santiago de Querétaro Qro. 76010, Mexico; miguel.contreras@uaq.mx; 6 Facultad de Ciencias Físico-Matemáticas, Universidad Autónoma de Sinaloa, Av. De las Américas y Blvd. Universitarios, Cd. Universitaria, Culiacán Sin. 80000, Mexico; carlos.duarte.galvan@uas.edu.mx

**Keywords:** embedded system, footprint measurements, Staheli arch index, Clarke’s angle, Smirak-Chippaux index, biomedical image processing

## Abstract

Manual measurements of foot anthropometry can lead to errors since this task involves the experience of the specialist who performs them, resulting in different subjective measures from the same footprint. Moreover, some of the diagnoses that are given to classify a footprint deformity are based on a qualitative interpretation by the physician; there is no quantitative interpretation of the footprint. The importance of providing a correct and accurate diagnosis lies in the need to ensure that an appropriate treatment is provided for the improvement of the patient without risking his or her health. Therefore, this article presents a smart sensor that integrates the capture of the footprint, a low computational-cost analysis of the image and the interpretation of the results through a quantitative evaluation. The smart sensor implemented required the use of a camera (Logitech C920) connected to a Raspberry Pi 3, where a graphical interface was made for the capture and processing of the image, and it was adapted to a podoscope conventionally used by specialists such as orthopedist, physiotherapists and podiatrists. The footprint diagnosis smart sensor (FPDSS) has proven to be robust to different types of deformity, precise, sensitive and correlated in 0.99 with the measurements from the digitalized image of the ink mat.

## 1. Introduction

It is well known that accurate and quantitative measurements are critical when a clinical diagnosis is given. Furthermore, manual measurements of the anthropometry of the foot can lead to wrong estimations because of the subjectivity involved in this task or the training and experience of the person responsible for the measurements. The diagnosis of foot deformities in individuals requires multiple measurements from the footprint to calculate a set of parameters that determine characteristics presented in the arch [1]. Studies have shown an inconsistent performance of measurements of the foot with traditional instruments, like a caliper or an ink mat [2,3,4]. When the orthopedist gives an incorrect diagnosis, the appropriate treatment will not be given in time and, consequently, it would affect the health of the patient. In order to be able to track the improvement of the patient over time and check the effectiveness of the prescribed treatment, it is necessary that measures be quantitative, consistent and precise every time. One of the most common deformities in the foot is the flat foot or *pes planus*. Flat foot is a condition where the medial longitudinal arch of the foot collapses causing partial or complete contact with the ground that will cause severe problems in the patient [5]. 

Conventional methods used in the clinical environment involve the use of a caliper by the orthopedist to estimate the required measurements of the foot. It is rudimentary and exhaustive to manually take all the measures and the variability, produced by the lack of precision which will lead to errors in the calculated parameters. There are several methodologies proposed for the computer-aided diagnosis of foot deformities. For example, Navarro et al. [6] designed a special sole template with pressure sensors to evaluate the distribution of the plantar pressure in different key parts. It is a good approach; however, it is necessary to manufacture different sizes of sole templates to adapt the position of the sensors to specific foot sizes in order to obtain precise measures in the appropriate areas. Hamza et al. in 2015 [7] used an ultrasonic sensor that passes under the foot and acquires the height of the medial longitudinal arch. Nonetheless, the use of ultrasonic sensors to measure the footprint in a straight line will ignore the measurements of different areas, not present in the arch, which could be substantial when a diagnosis is given. On the other hand, There are several computational techniques that are responsible for reconstruction of the footprint; for example, Guerrero-Turrubiates et al. [8] proposed the use of a parabola detection algorithm in the areas of the arch and the heel of the foot, also the finite element method are widely used to reconstruct the footprint, even the complete foot or the pressure caused by itself [9,10,11]. Both providing excellent results but with a high cost in terms of computing resources and time. It is very important to consider the required resources when implementing the algorithm, especially when it is necessary to generate an application for embedded devices or dispense with a high-performance and expensive computer. A recent study [12] presented a system for the analysis of the foot arch by using an RGB-D camera for 3D image reconstruction of the footprint; such system has shown good reliability in the performed experiments. However, the measurements of the footprint and the relation of the forefoot and the barefoot with the midfoot are left behind since they only focus on the analysis of the foot arch. Consequently, the complete analysis of the footprint cannot be given and this could potentially generate an insufficient diagnosis. Currently, there are no RBG camera-based sensors that automatically analysze the measurements of the footprint. Table 1 shows a comparison between the methods described above and our current approach (FPDSS).

**Figure 1:**
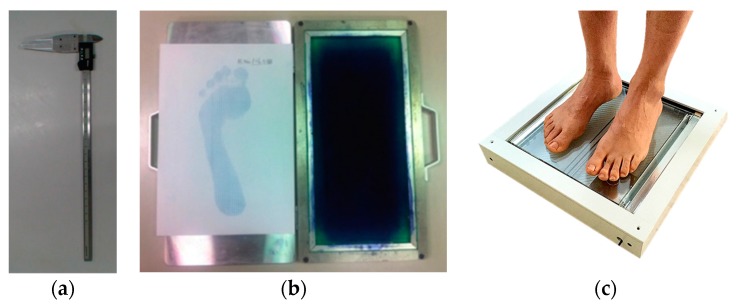
Traditional devices used for the measurement of the foot: (**a**) Digital caliper; (**b**) Ink mat; (**c**) Digital podoscope (modified from [3]).

There are some devices in the market that provide digital images of the footprint but only at high pressure areas. Additionally, it is necessary for the user to enter quantitative measurements of the foot. These problems do not allow the automatic calculation of parameters required for the diagnosis of deformities in the footprint. In Figure 1, the conventional devices used in the diagnosis by the orthopedist can be observed. The data obtained from the digital caliper and the ink mat will always result in measurements that are sensitive to the training of the orthopedist because the area where the measurement must be made may vary from one patient to another [2]. Computerized podoscope (Plantoscopio Computarizado, Puebla, Mexico) is an optical device with which it is possible to scan and visualize the footprint without any analysis that determines the condition present in the foot. In the same way, ArcoScan (CiPar Ingeniería, Paraná, Argentina) is a podoscope that allows you to digitalize and store the footprint but, with the difference to the previous one, it also allows the estimation of areas with higher pressure by using the intensity levels of the pixels in the region of the foot. However, these devices do not include a qualitative or quantitative analysis of the foot, like measurements and angles of the footprint, and they will always require the interpretation by the orthopedist in order to give a diagnosis. Having measurements on a real scale and the automatic calculation of parameters that indicate deformities in the foot is very important to provide more efficient diagnosis. It is important to mention that foot deformities should be treated as quickly and efficiently as possible to reduce the effects they may cause in other areas of the body [13,14,15]. When the deformity is correctly diagnosed, an appropriate treatment is given to the patient.

However, this process can be automatized using a digital footprint analysis smart sensor, which would considerably take out all the disadvantages of manual measurement while computing the parameters needed for the correct diagnosis. The objective of this article is to introduce a sensor that integrates the capture of the plantar footprint, the analysis of the image and the interpretation of the results through a quantitative evaluation. The advantage over conventional methods lies in the ability to automatically detect footprint measurements and the angle of the longitudinal arch of the foot in a real scale quickly and with minimal computational resources and, in the same way, the calculation of three essential parameters to fully describe the footprint quantitatively: Staheli index, Clarke’s angle and Smirak-Chippaux Index. Consequently, it provides an accurate interpretation of the results for both, the diagnosis and the orthopedist. Finally, the calculated parameters from the patient’s footprint are saved and plotted to graphically observe the improvement over time to ensure that the treatment given by the orthopedist is right for the condition, so the patient’s health is not to put at risk and the recovery time is reduced. A smart sensor is presented for the automatic quantitative interpretation and analysis of footprint images resulting in a computer-aided diagnosis of deformities in the foot.

## 2. Materials and Methods 

### 2.1. Smart Sensor Setup

**Figure 2:**
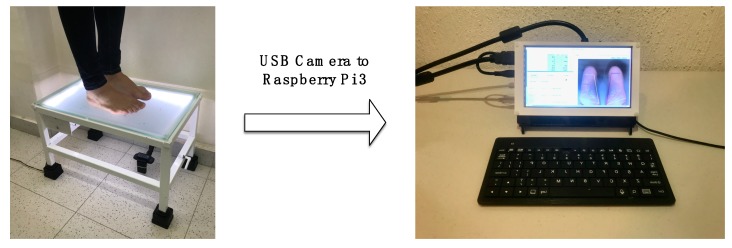
Adaptation of the original podoscope placing a frosted film on the top and the camera connected to the Raspberry Pi 3 via USB. Using an USB camera, the Raspberry Pi 3 acquires an image of the patient’s footprints (**left**) and process it to display the results in the graphical user interface (**right**).

**Figure 3:**
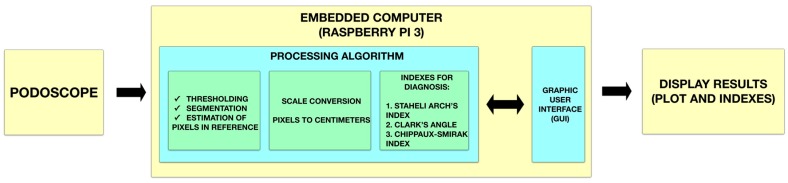
Block diagram of the smart sensor.

The implementation of the smart sensor presented required the use of a modified regular podoscope, as we can observe in Figure 2; a one squared centimeter black sticker as a reference area was placed on the top of a tempered glass with a frosted film [13] and a generic camera was embedded under the glass, in this case, a Logitech HD Pro Webcam Model C920 (Logitech, Lausanne, Switzerland) was used. This allowed the capture of the footprint in an image file for the computer. The parameters for image capture of the camera were set to 40% brightness, 50% contrast, 60% sharpness and 60% of the white balance. As shown in Figure 3, the camera was connected to an embedded computer (Raspberry Pi 3, Raspberry Pi Fundation, Cambridge, UK) with the Raspbian Jessie Operative System for the analysis of the obtained images through an interaction of the processing algorithm and the input of the user in the graphical interface. 

The smart sensor and the involved process can be applied in any generic podoscope by integrating a camera to take the images of the footprint and using the software for analysis. In this way, conventional equipment can be improved to provide more accurate diagnostics. Figure 3 shows the process that the smart sensor follows to provide the results to the user.

### 2.2. Footprint Parameters

Three parameters were used for the analysis and evaluation of the FPDSS images and obtained data. Therefore, this section explains the importance of the metrics used and how they are obtained in footprint images.

The Staheli arch index is used to describe the relation between the minimum width of the midfoot and the maximum width of the hindfoot. This index has been one of the most studied parameters for the description of footprint deformities and historically represents an important index for the detection of flat foot [16]. In addition to the above, it is considered as an essential diagnostic method compared to the talar—first metatarsal angle, commonly used by specialists in the field for the detection of deformities in the footprint [17].

Clarke’s angle or arch angle describes the measure of the internal longitudinal arch. According to [18], this parameter may have been the first proposed for studies in deformities and posture, as it is proportional to the arch height. This index comes from the angle between a line that goes from the outer point of the forefoot to the outer point of the hindfoot and another line connecting the outer point of the forefoot and the inner point of the midfoot.

**Figure 4:**
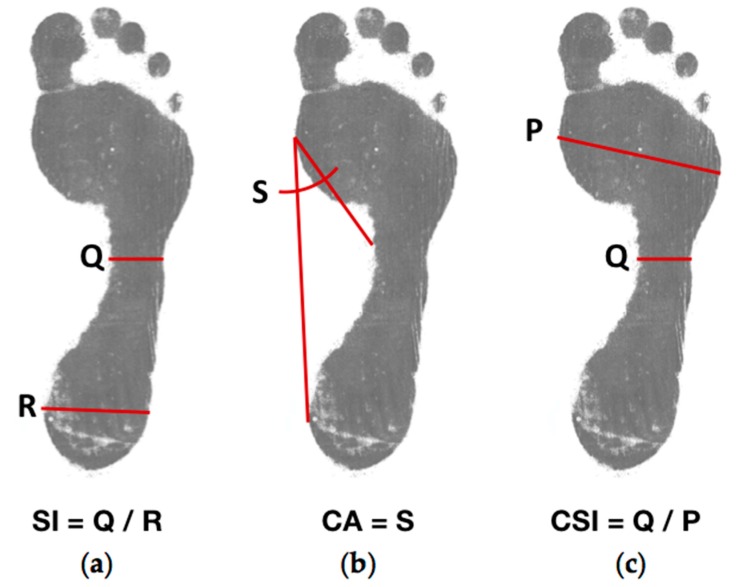
Footprint parameters used by the FPDSS to evaluate the deformities in the footprint: (**a**) Staheli index; (**b**) Clarke’s angle; (**c**) Chippaux-Smirak index.

Finally, the Chippaux-Smirak index is the relation between the minimum width of the midfoot and the maximum width of the forefoot. When the arch of the foot is larger, this index decreases its value [18]. By using different parameters that entirely describe the footprint, the values obtained during the analysis allow a solid diagnosis performed by the FPDSS. Figure 4 describes the lines mentioned above for the calculation of the different parameters.

### 2.3. Algorithms for Estimation of Measures in Footprint

The proposed algorithm is based on the application of image processing techniques. The programming language used for the development of this algorithm was C. The design of a graphical user interface (GUI) was performed with the use of GTK+ for a friendly user interaction with the results and the algorithm. These methods were implemented using the OpenCV library [19] for the manipulation of the footprint images. 

In the first part, the camera acquires an 8-bit image in JPEG format with dimensions of 1920 × 1080 pixels and RGB color space. Once the image is introduced in the software, several preprocessing steps are made. Firstly, the RGB color image was converted into CIELAB (CIE 1976 L*a*b*) color space. CIELAB provides a better interpretation of the image for the algorithm since the luminosity is separated in a single channel and totally ignored in the color channels (a* and b*). The output channels of the CIELAB model gave a better correlation with the visual interpretation of the footprint images, something that is of great importance when the area of the footprint needs to be separated from the skin of the foot. After that, a range of values in the a* and b* channel was tested for binarizing the image based on an image thresholding step. The thresholding for a* channel was set to 122 and for b* channel was set to 131, since that combination of thresholds correspond to any intensity of the yellow and red color in the CIELAB color space. That means that any intensity above the thresholds will be set as white pixels (1) and the rest will be black pixels (0), the range of intensity per pixel goes from 0 to 255 in 8-bit images. Subsequently, the region of the footprint is correctly segmented in a binary image. Then, the binarized image of the previous step is dilated to expand the size of the footprint and to cover all the small parts that remained outside the foot during the thresholding process. Finally, the image is segmented only to get the area that we are interested in; that is to say, the footprint excluding the toes. This was performed using a function that detects the contour of the biggest object in the image and traces a boundary so that everything outside the contour is set as background or black pixels.

After the preprocessing stage of the footprint is done, the algorithm looks for the one square centimeter reference in the R (red) channel of the image, since it is the channel of the RGB color model where it is easier to obtain the reference. This is accomplished with a function that iterates over the image and looks for continuous black pixels in the bottom and counts them to know the area of the square [20]. The number of pixels in one square centimeter is saved for using it when a conversion from pixels to centimeters is requested.

Afterwards, the algorithm finds the key points of three parameters in the processed image used for the analysis and detection of deformities in the footprint [1,21]. The parameters automatically determined by this algorithm are the Staheli arch index, Clarke’s angle and the Chippaux-Smirak index. 

**Figure 5:**
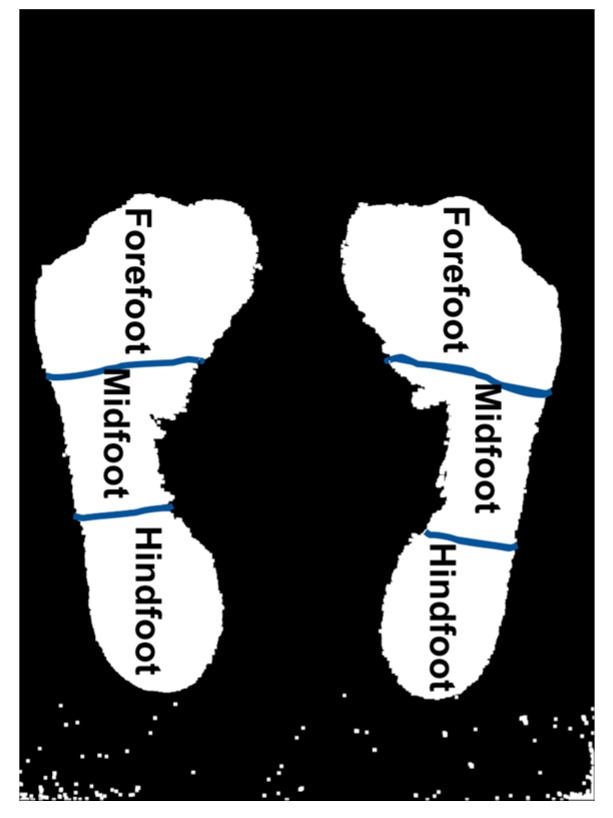
Divisions of the footprint in 3 zones.

For the calculation of the parameters, the foot is divided into three main areas: hindfoot, midfoot and forefoot as shown in Figure 5. Before finding the parameters in the footprint, a function is implemented to find if there is a right foot, left foot or both by iterating over the binarized image to find the number of contours and their corresponding position. By knowing which foot is on the image, the right functions are applied and, therefore, it maximizes the precision of the calculation and diagnosis. Finally, the algorithm first obtains the measurement of the line in pixels, by using the location of the pixels at the starting point and the ending point with Equation (1), and then transforms it to centimeters using the previously calculated reference:

(1) $$Z\;=\;\sqrt{{\left(x_{2}-x_{1}\right)}^{2}+{\left(y_{2}-y_{1}\right)}^{2}}$$

**Figure 6:**
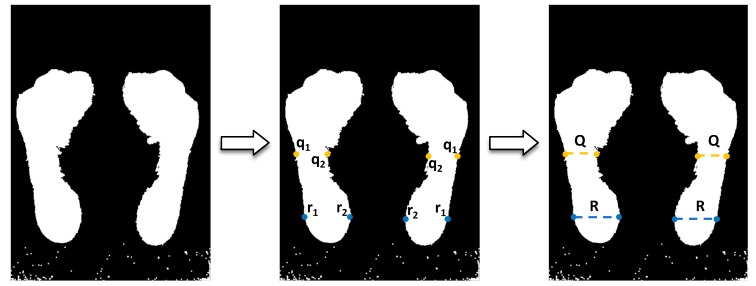
The process of the Staheli arch index calculation.

The Staheli arch index was estimated by the division of the narrowest line in the midfoot (Q) over the widest line in the hindfoot (R) [16]. The narrowest line in the midfoot is calculated from a function that iterates row by row from right to left (for left foot) or left to right (for right foot), and it finds the pixel that is further from the starting point (inner point or q2). Then, it draws a horizontal line that goes from that point to the end of the segmented footprint (q1) at the left (for left foot) or right (for right foot). For the widest line in the hindfoot, the function is the same as above, but this time it finds the pixel that is the closest one from the starting position of the algorithm (outer point or r2) and the point (r1) at the end of the horizontal line through the hindfoot. Figure 6 illustrates the process described above.

**Figure 7:**
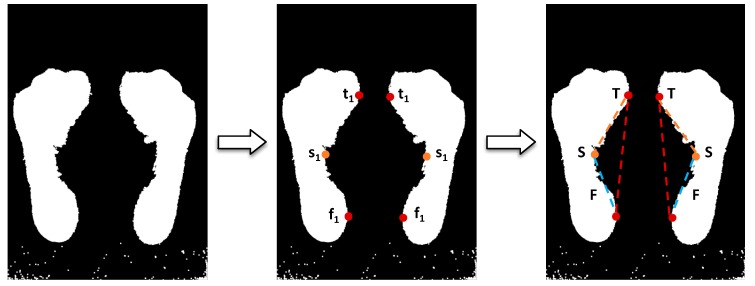
The process of Clarke’s angle estimation.

The Clarke’s angle is calculated by taking the angle between a line T, that goes from the outer point of the forefoot to the outer point of the hindfoot, and a line S, that goes from the outer point of the forefoot and the inner point of the midfoot [22]. The line T is calculated by doing an iteration, in the forefoot and hindfoot, pixel by pixel from right to left (for left foot) or left to right (for right foot) to find the pixel (t1) that is the closest to the starting point in both areas. The line S is calculated by drawing a line from the outer point in the forefoot (t1), that was found in the previous step, to the inner point of the midfoot (s1). After drawing and estimating the measurement of both lines, the angle estimation requires a measurement of a third line, F, that goes from the outer point of the hindfoot (f1) to the inner point of the midfoot (s1). Figure 7 shows the lines specified above. Equation (2) comes from the calculation of the angle of an isosceles triangle and it is used for Clark’s angle estimation in this analysis:

(2) $$\mathrm{Clark}’s\;\mathrm{Angle}\;=\;\cos^{-1}\left(\frac{S^{2}-T^{2}-F^{2}}{-2\;T\;F}\right)$$

**Figure 8:**
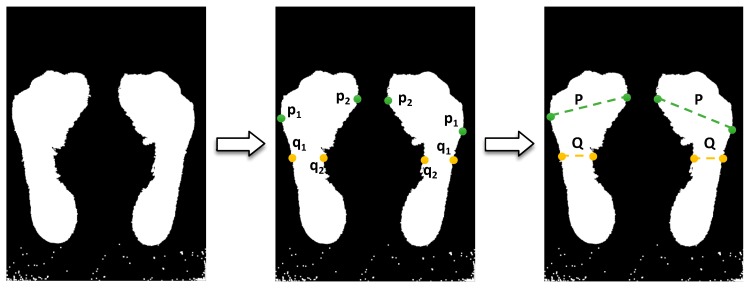
Process of the Chippaux-Smirak index calculation.

The Chippaux-Smirak index requires the measurement of the widest line (P) of the forefoot because we already have the narrowest line (Q) in the midfoot from the Staheli arch index calculation. The algorithm iterates over the forefoot to find the widest line using outer (p1) and inner (p2) points from the starting position of the algorithm (middle of the image), as shown in Figure 8. After having calculated the measurements of both lines, the index is estimated by Equation (3) [18]:

(3) $$\mathrm{Chippaux}-\mathrm{Smirak}\;\mathrm{index}\;\left(\%\right)\;=\;\frac{Q}{P}\times 100$$

**Figure 9:**
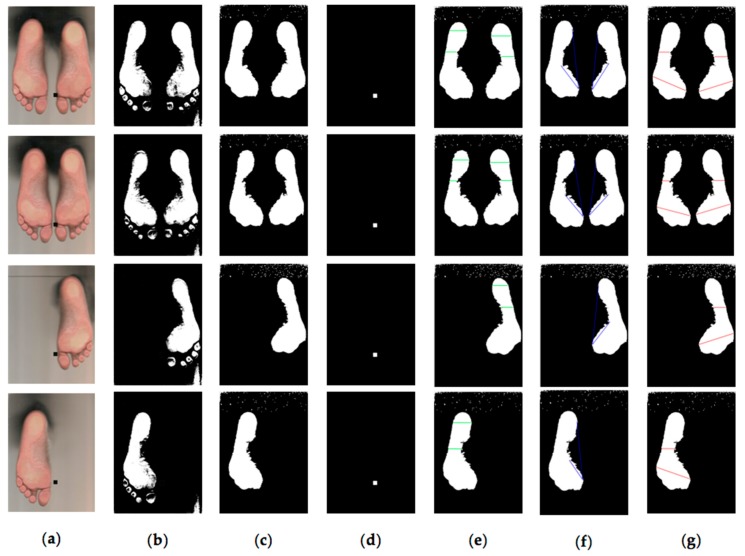
Steps of the algorithm for the analysis of the footprint: (**a**) RGB image obtained from podoscope; (**b**) Thresholding at 183 of red channel; (**c**) Segmentation of the footprint and removal of toes; (**d**) Location of the reference (square centimeter) in the image; (**e**) Staheli arch index; (**f**) Clarke’s angle; (**g**) Chippaux-Smirak index.

Finally, the algorithm saves the acquired parameters information for further analysis and gives the possibility to add new lines in the image with an automatic calculation of the distance in centimeters. In Figure 9, the results of each part of the algorithm are shown visually. 

### 2.4. Graphic User Interface

The graphic user interface (GUI) was developed to provide a friendly tool for the user to quantitative analyze and measure the footprint images by implementing the proposed algorithm. The first step consists in using the camera for acquiring the footprint image and, once it is selected, the algorithm processes the image and gives the output by automatically drawing the aforementioned lines, with their respective measurements in centimeters, and calculating the three parameters. 

**Figure 10:**
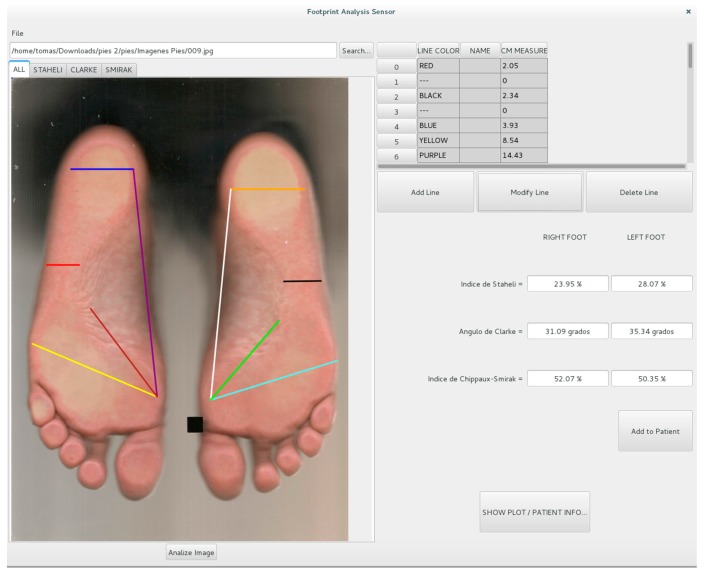
The graphical user interface of the footprint analysis sensor.

As shown in Figure 10, the GUI has some extra functions that give the user the possibility to add new lines, or modify and remove lines that are already drawn in the image. This is performed because the image is placed in the bottom of a drawing area where the user can take manually measurements using a mouse. The software records the coordinates of the starting point and the ending point of the new line so the conversion from pixels to centimeters can be performed. Also, it recognizes when a click is given in a specific line so it can be either modified or erased. There are four tabs on the top of the image; each one includes different lines drawn by the software. For example, the first one includes all the lines that the algorithm used for the detection of the three parameters. The rest of the tabs include just the lines used for the estimation of the index indicated in its own title.

**Figure 11:**
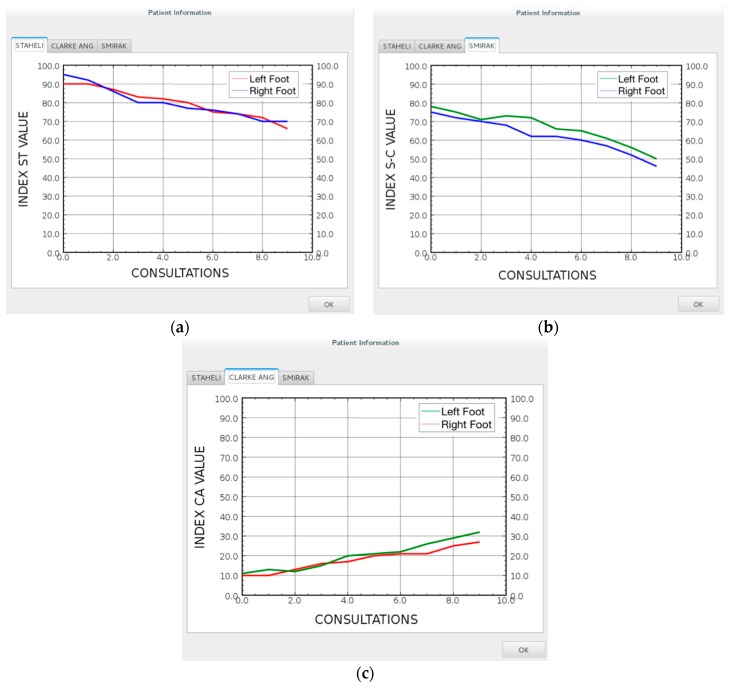
An example plots of a patient improvement over time with the evaluation of different parameters: (**a**) Staheli Index; (**b**) Chippaux-Smirak Index; (**c**) Clarke’s Angle.

The parameters that result from the analysis of the footprint can be plotted and saved over time to graphically observe the improvement of the patient, as depicted in Figure 11, and the effectiveness of the prescribed treatment. There are three plots, one for each index, and the index can be observed for each foot independently. Finally, the user has the possibility to export as PDF or print the image with the drawn lines. 

### 2.5. Experimental Setup

Precision and accuracy experiments were designed to evaluate the robustness and reliability of the proposed algorithm. The experiments were performed in 15 women and 25 men between the ages of 18 and 24 that presented different types of foot; cavus foot, normal foot and flat foot. Automatic measurements were implemented using our designed software and manual measurements required a manually quantitative interpretation of the ink mat.

The precision experiment consisted in asking the subjects to step on the podoscope, in a bipedalism posture, so that we could acquire the contact region of the feet with the glass using the camera. Next, the subject stepped off the podoscope. This process was repeated five times so that we could obtain the enough number of images per subject to evaluate the precision of the algorithm. We collected 200 images of footprints from the subjects with their respective calculation of parameters for each foot.

In addition, the accuracy experiment requires the use of a reference measurement to compare the calculated parameters from the footprint images. Therefore, the ink mat was used to perform manual measurements as a reference for our method. After the footprint images acquiring process, we asked the subject to place the foot individually on the ink mat. Then, we got the footprint on a sheet of paper where we could calculate the manual measurements of the parameters obtained by our software. In order to get the most comparable data and to address the human error from manual measurements, we also processed the footprints from the ink mat using our algorithm. 

## 3. Results

Once the analysis was performed in the collected images, we were able to compare the calculated parameters obtained using our proposed method. For each subject, a precision analysis was performed using the one-sigma and three-sigma rule, as described in [23], which tells us that 99.7% of the data must fall within three standard deviations of the mean to test the distribution normality of the dataset [24]. 

**Figure 12:**
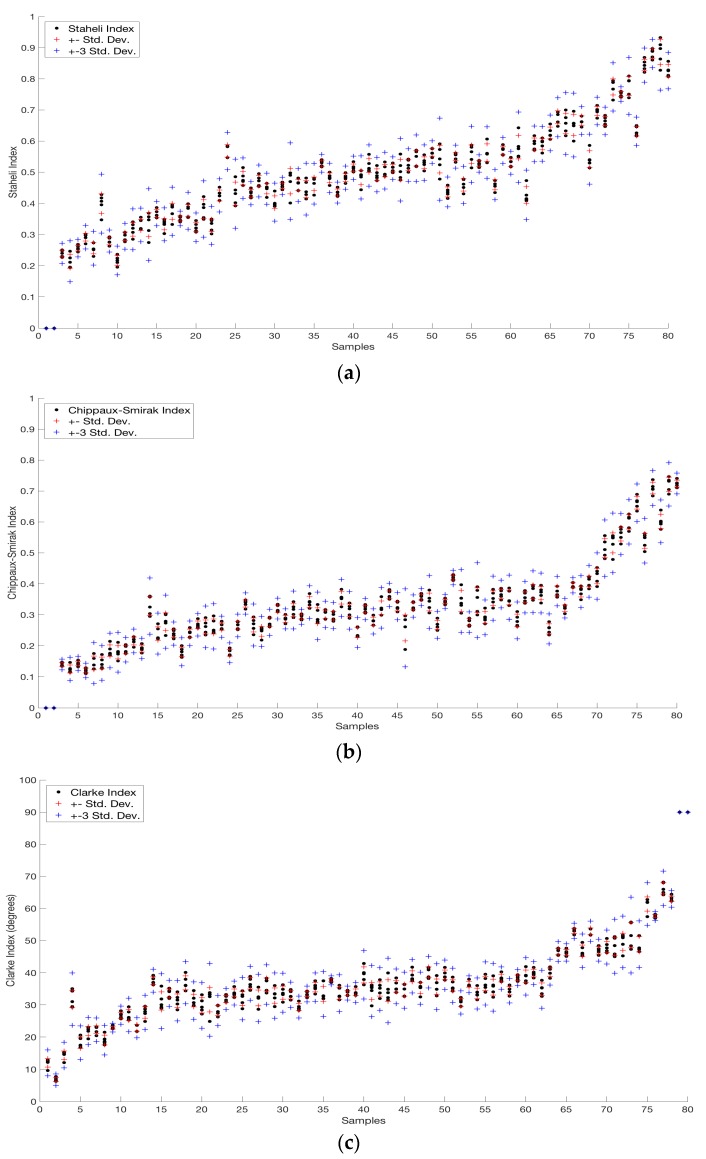
Precision analysis results where the vertical axis represents the value of the index and the horizontal axis is the right and left foot of the subjects arranged two by two: (**a**) Staheli index; (**b**) Chippaux-Smirak Index; (**c**) Clarke’s Angle.

The mentioned rule was applied on every index (Staheli, Chippaux-Smirak and Clarke index) of a dataset of five images per subject’s foot (40 subjects), as we can observe in Figure 12; where the obtained data from both feet has been sorted in an ascending way according to the value of the right foot of the subject and placing the value of the left foot next to it. All data have satisfied the statistical analysis showing the robustness of the implemented algorithm.Specifically, the analysis of the one-sigma rule tells us that approximately 68% of the data must lie between one standard deviation from the mean of a set of data. This was performed on data and the results are presented in Table 2, which shows the number of cases with a specific amount of values that are outside of the analysis of one-sigma per every subject’s foot. Since there were no cases with more than three values outside of the one-sigma evaluation, we chose to include only 2, 1 and 0 cases in the table. 

In order to perform a complete analysis of the accuracy of our method, three large sets of data per index were compared; the data acquired by the proposed method, data from the manual measurements in the footprints obtained from the ink mat on a sheet of paper, and the data obtained by applying our algorithm on the digitalized footprint of the ink mat. To easily visualize and compare the data, the acquired footprints were classified into five groups, as observed in Table 3, based on the limits established in [25,26,27]: cavus foot, cavus-normal foot, normal foot, normal-flat foot and flat foot. 

**Figure 13:**
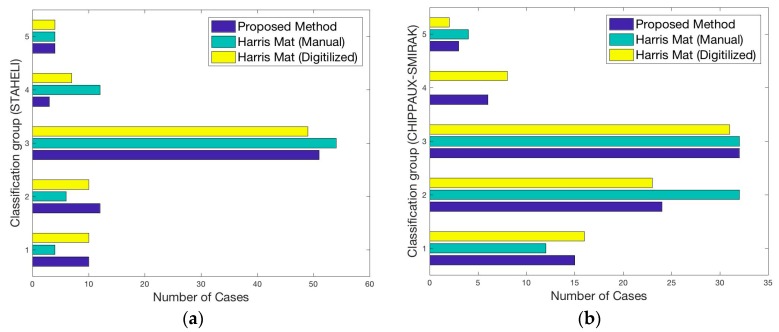

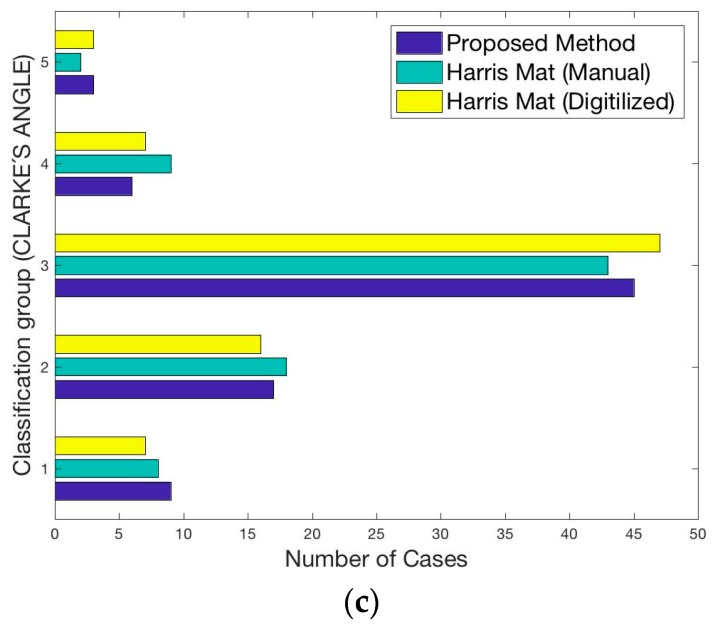
Accuracy analysis performed on three different datasets (proposed method, manual ink mat and digitalized ink mat). The vertical axis represents the five groups of classified footprints. The horizontal axis gives the number of cases in the respective group: (**a**) Staheli index; (**b**) Chippaux-Smirak Index; (**c**) Clarke’s Angle.

The comparison of the three datasets for each index is shown in Figure 13. Furthermore, the relationship between the three methods was verified using the Pearson correlation coefficient, so we could obtain and compare the accuracy and reliability of the proposed method among the others [28]. Table 4 indicates the results of the correlation analysis.

## 4. Discussion and Conclusions

Through the analysis of the data obtained in the performed experiments, we can infer the subjectivity of manual measurements of the footprint and the respective calculation of three major parameters: Staheli, Chippaux-Smirak and Clarke index, thus, our proposed smart sensor for measuring and diagnosing deformities in the footprint through image processing eliminates the subjectivity of manual diagnosis. Additionally, it performs a quantitative analysis and presents a standard measurement for the correct diagnosis and treatment of the patient. For that reason, the quantitative interpretation gives a more accurate perspective of the medical condition of a patient, because the smart sensor records the history of parameters over consultations to evaluate the effectiveness of the treatment. The mentioned advantages in the diagnosis using the implemented method are not present in any of the current methods for footprint analysis. Although the experiments were conducted on people within the age range of 17–24 years, they were carefully selected to encompass the different footprint classification groups.

The precision analysis shows the reliability and consistent of the proposed smart sensor since all measurements for every subject stay in the specified range of 3 standard deviations from the mean. This confirms the distribution normality of the data proving that the calculated measurements for any subject are obtained with consistency and without significant variation. In addition, the accuracy analysis shows the difficulties of manual calculation of the parameters from footprints obtained using the ink mat, where results depend on the experience and perception of the technician performing them. At the same time, the ink mat can lead to errors since the ink placed on the paper can take a larger area than expected, leading to significantly differences when compared to others methods as stated in [29]. The implemented method indicates that the measurements are correlated in 0.99 when compared to a digitalized version of the footprint from an ink mat. Consequently, we were able to correctly detect and keep track of abnormalities of the footprint by the Staheli, Chippaux-Smirak and Clarke Index in 40 subjects using our proposed method of image processing and a modified podoscope.

First, a comparison between the results of the implemented method and the manual measurements was made and we found an interesting disparity in the data. After that, the footprint sheets from the ink mat were scanned and the implemented algorithm was applied, resulting in an improved correlation of our results and the scanned footprints since the average error at classifying them went from 21% to 8%. As verified by several studies [2], the standardization of measurement techniques is required to reduce the inter-observer and random errors, something that it is hard to achieve when different people manually calculate specific parameters of the footprint. Recently, Su et al. in 2016 [30] described the estimation of plantar pressure and arch index through the intensity of the pixels from a digitalized version of the footprints from an ink mat but still using a qualitative interpretation of the obtained data. At the same time, others [12] have proposed a solution for the footprint deformities using an RGB-D camera for reconstruction and performing a quantitative analysis from the foot arch to obtain several arch parameters.

In this study, a footprint diagnosis smart sensor was proven to be robust to different types of deformity, precise, sensitive and adaptable to most podoscopes used by specialists. In consequence, the possibility of performing a quantitative analysis through the presented smart sensor allows the operator to carry out a statistical control of the patient over consultations, resulting in an evaluation of the effectiveness of the prescribed treatment. Additionally, the low computational cost of the implemented algorithm allows it to be used in embedded devices like a Raspberry Pi. Above all, this smart sensor will ultimately result in an accurate quantitative diagnosis that will significantly improve the patient’s condition in less time using the proper treatment. Further research is needed to correlate the footprint deformations on a bi-pedestation and mono-pedestation position and, therefore, perform an analysis of the postural effects in the footprint.

## Figures and Tables

**Table 1:** Comparison of the methods used by different sensors to diagnose foot deformities.

Authors/Method	Objective of the Method	Type of Sensor	Accuracy
Navarro et al. [6]	Detection of flat foot using estimation techniques	Pressure sensors	N/A ^1^
Hamza et al. [7]	Flatfoot detector using the height of the arch	Ultrasonic sensors	100% in 20 subjects
Guerrero-Turrubiates et al. [8]	Detection of parabolas dimensions in the footprint	Footprint scanner	N/A ^1^
Chun et al. [12]	3D image reconstruction of the footprint	RGB-D camera	~97.32% in 11 subjects ^2^
FPDSS	Detection of deformities in the footprint through image processing	RGB camera with reference	99.38% in 40 subjects ^2^

^1^ Accuracy not specified in article. ^2^ Mean of the accuracy in the three parameters calculated by the sensor.

**Table 2:** Number of cases with the respective amount of values (2, 1 or 0) out of the one-sigma (1σ) rule in every calculated index from the dataset of a subject’s foot.

Index Name	Amount of Values out of 1σ Rule		
	2	1	0
Staheli Index	14	57	9
Chippaux-Smirak Index	18	46	16
Clarke Index	12	49	19

**Table 3:** The range for the classification into five groups of footprints whereas: 1—Cavus foot; 2—Cavus-Normal foot; 3—Normal foot; 4—Normal-Flat foot; 5—Flat foot.

Index Name	Classification Groups				
	1	2	3	4	5
Staheli Index	<0.3	0.3–0.4	0.4–0.7	0.7–0.8	>0.8
Chippaux-Smirak Index	<0.22	0.22–0.3	0.3–0.5	0.5–0.7	>0.7
Clarke Index	>50	38–50	26–38	15–26	<15

**Table 4:** Comparison of values obtained from the evaluated methods and their respective correlation coefficient.

Compared Methods	Correlation Coefficient ^1^
Ink mat (manual) vs. Ink mat (digitalized)	0.9618
FPDSS ^2^ vs. Ink mat (manual)	0.9619
FPDSS ^2^ vs. Ink mat (digitalized)	0.9938

^1^ Calculations based on the method of Pearson correlation coefficient [28]. ^2^ FPDSS = Footprint Diagnosis Smart Sensor.
